# Pharmacokinetics of loxoprofen in a self-administered overdose in a Japanese patient admitted to hospital

**DOI:** 10.1186/s40780-021-00216-9

**Published:** 2021-09-07

**Authors:** Koichiro Adachi, Yuki Sugitani, Ryo Unita, Kosuke Yoshida, Satoru Beppu, Mariko Terashima, Masaya Fujii, Makiko Shimizu, Hiroshi Yamazaki

**Affiliations:** 1 Laboratory of Drug Metabolism and Pharmacokinetics, Showa Pharmaceutical University, 3-3165 Higashi-tamagawa Gakuen, Machida, Tokyo, 194-8543 Japan; 2 Kyoto Medical Center, Fushimi-ku, Kyoto, 612-8555 Japan; 3 Present address, Himeji Medical Center, Himeji, Hyogo 670-8520 Japan

**Keywords:** Pharmacokinetic modeling, Overdose, Absorption, Elimination

## Abstract

**Background:**

Loxoprofen is a propionic acid derivative and is the most widely prescribed non-steroidal anti-inflammatory drug in Japan. Loxoprofen is generally considered to be relatively nontoxic.

**Case presentation:**

A 33-year-old man (body weight, 55 kg) who intentionally took an overdose of 100 tablets of loxoprofen (6000 mg) as a suicide attempt was emergently admitted to Kyoto Medical Center. On arrival, the patient was suffering disorders of consciousness. His plasma concentrations of loxoprofen and its reduced *trans-*alcohol metabolite were 52 and 24 μg/mL, 3.7 and 2.3 μg/mL, 0.81 and 0.54 μg/mL, and 0.015 and 0.011 μg/mL, respectively, at 4, 26, 50, and 121 h after the oral overdose. The observed apparent terminal elimination half-life of loxoprofen during days 1 and 2 of hospitalization was in the range 6–12 h, which is several times longer than the reported normal value. This finding implied nonlinearity of loxoprofen pharmacokinetics over the current 100-fold dose range, which could affect the accuracy of values simulated by a simplified physiologically based pharmacokinetic (PBPK) model founded on data from a normal dose of 60 mg. The reasons for the delayed eliminations from plasma of loxoprofen and its *trans-*alcohol metabolite in this case are uncertain, but slight renal impairment (low eGFR values) developed on the second and third hospital days and could be a causal factor.

**Conclusions:**

Because the patient’s level of consciousness had gradually improved, he was discharged on the fourth day of hospitalization. The virtual plasma exposures of loxoprofen and its reduced *trans*-alcohol metabolite estimated using the current simplified PBPK model were lower than the measured values in the overdose case. The present results based on drug monitoring data and pharmacokinetic predictions could serve as a useful guide in cases of loxoprofen overdose.

## Background

Loxoprofen, a propionic acid derivative, acts as a cyclooxygenase inhibitor and has been in clinical use since 1986 in the world [1, 2]. Loxoprofen is currently the most prescribed non-steroidal anti-inflammatory drug (NSAID) in Japan (30% of ~ 2,000,000 patients using NSAIDs in 2017 [3]). Loxoprofen is a prodrug that is reduced to its active metabolite, the *trans-*alcohol form, by carbonyl reductase enzymes in the liver [4]. Although loxoprofen is often considered to be relatively nontoxic, one case report suggested prolonged intrahepatic cholestasis in a woman after normal use for 5 days [5]. Also, a man who died as a result of an anaphylactic reaction had a postmortem blood loxoprofen concentration of 1.2 μg/mL, which is within the therapeutic range [6]. The monitoring of plasma concentrations of loxoprofen may be considered in clinical or emergency situations.

The drug monitoring of steady-state plasma concentrations of individual patients in the clinical setting can be supported by pharmacokinetic models and simulations. Full physiologically based pharmacokinetic (PBPK) models [7] can predict drug monitoring results in patients [8–10]. However, we have developed simplified PBPK models [11] and have applied these models to cases of edoxaban overdose [12] and a combined overdose of duloxetine with other antipsychotic drugs [13]. The practical application of such PBPK models is suggested for paramedical staff in emergency clinical practice [12, 13].

## Case presentation

Here, we describe the case of a 33-year-old man (body weight, 55 kg) who intentionally took an overdose of 100 tablets (6000 mg) of loxoprofen (usual clinical dose in the range 60–180 mg/day [1, 2]). On arrival at Kyoto Medical Center, with empty heat seals for loxoprofen, the patient was suffering disorders of consciousness. His awareness level was assessed, and the Glasgow Coma Scale score was eye 1, verbal 1, and motor 1 (E1V1M1). Contralateral light reflexes were bilateral 2/2 (−/−), the blood pressure was 150/90 mmHg, the heart rate was 106 bpm, the respiratory rate was 18/min, and the body temperature was 36.7 °C. No rigidity of the arm and no snore-like breathing were noted in an arm drop test. The following tests all gave negative results: drug intoxication (Triage DOA, Sysmex, Kobe, Japan), head computed tomography, magnetic resonance imaging, spinal fluids, and simple electroencephalography. After the patient’s level of consciousness had gradually improved, subsequent questioning revealed that he had taken a high dose of loxoprofen but had not consumed any alcohol. The family of the patient was also interviewed regarding the circumstance of overdoses. No history of asthma, hepatic dysfunction, impaired renal function, or autoimmune disease or a family history of autoimmune disease was noted. The clinical laboratory results for the current patient are shown in Table 1. The patient developed slight renal impairment (low eGFR values) on the second and third days of hospitalization, but was discharged on the fourth day of hospitalization.

**Table 1:** Clinical laboratory results for a patient who took a single oral overdose of 6000 mg loxoprofen

	Day 1	Day 2	Day 3	Day 6
Aspartate aminotransferase (U/L)	45	39	46	32
Alanine aminotransferase (U/L)	30	27	29	28
eGFR (mL/min/1.73 m2)	77.0	62.8	68.9	90.8
Serum creatinine (mg/dL)	0.93	1.12	1.03	0.80
Creatinine clearance (mL/min)	87.9	73.0	79.4	102.0

**Fig. 1:**
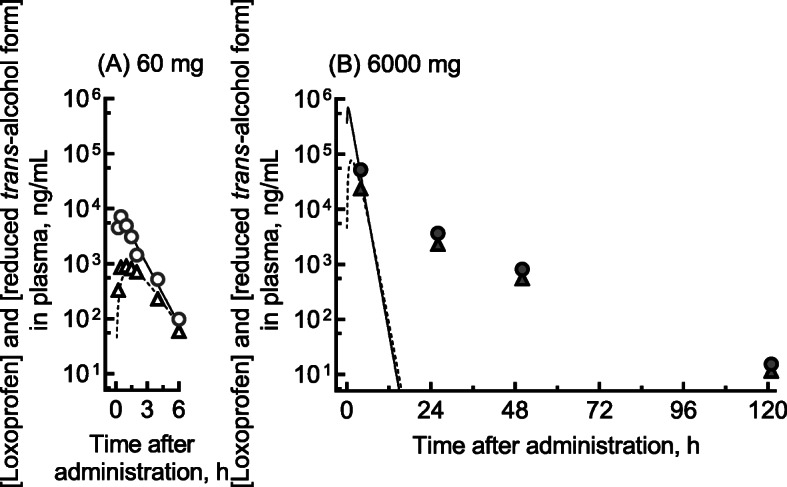
Measured (plots) and estimated (lines) plasma concentrations of loxoprofen and its reduced *trans-*alcohol metabolite: measured values were from the literature (**A**) and from the patient who took a single oral overdose (**B**). Plasma concentrations of loxoprofen (circles) and its reduced metabolite (triangles) were taken from the literature for 24 volunteers who were administered a therapeutic dose of 60 mg [4, 14] (**A**). Plasma concentrations of loxoprofen are also shown for the patient who took a single oral overdose of 6000 mg (**B**). The PBPK-modeled plasma concentration curves for loxoprofen (solid lines) and its reduced *trans-*alcohol metabolite (broken lines) after virtual administration of loxoprofen are also shown

We report herein the measured plasma concentrations of loxoprofen and its reduced *trans-*alcohol metabolite during 3 days of hospitalization and the PBPK-modeled concentration profiles of loxoprofen self-administered after a single oral overdose **(**Fig. 1B). The patient gave written informed consent to take part in this study and for its publication. The Ethics Committee of Kyoto Medical Center approved this study (18–018). Frozen plasma samples collected from the patient were pharmacokinetically analyzed. After samples were deproteinized with four volumes of acetonitrile, the plasma concentrations of loxoprofen and its reduced *trans-*alcohol metabolite were quantified by liquid chromatography using a gradient elution program followed by tandem mass spectrometry [13] according to the previously reported methods [4] with slight modifications. An API4000 tandem mass analyzer (AB Sciex, Framingham, MA, USA) was used in electrospray negative ionization mode and was directly coupled to a Shimadzu LC-20 AD system equipped with an octadecylsilane (C_18_) column (XBridge, 3.5 μm, 2.1 mm × 150 mm, Waters, Milford, MA, USA). The LC conditions were as follows: solvent A was 0.1% formic acid in water, and solvent B was 0.1% formic acid in acetonitrile. The following gradient program was used with a flow rate of 0.25 mL/min: 0.0–0.5 min, hold at 10% B; 0.5–21 min, linear gradient from 10% B to 50% B (v/v); 21–22 min, linear gradient from 50% B to 95% B (v/v); 22–25 min, hold at 95% B; and 25.1–30 min, hold at 10% B. The temperature of the column was maintained at 40 °C. Prepared samples (2.0 μL) were injected using an auto-sampler. Loxoprofen and its reduced *trans-*alcohol metabolite were quantified using *m/z* 245 → 83 and *m/z* 247 → 191 transitions, respectively. Loxoprofen-*d*_3_ and *trans-*loxoprofen-*d*_3_ alcohol were used as internal standards using *m/z* 248 → 83 and *m/z* 250 → 194 transitions, respectively. Under the present conditions, the plasma levels of loxoprofen and its reduced *trans-*alcohol metabolite were measurable at ≥10 ng/mL and detectable at ≥1.0 ng/mL. Authentic loxoprofen and its reduced *trans-*alcohol form were purchased from Fujifilm Wako Pure Chemicals, Osaka, Japan, and loxoprofen-*d*_3_ and the *trans-*alcohol form of loxoprofen-*d*_3_ were purchased from Toronto Research Chemicals, North York, ON, Canada. The measured plasma concentrations of loxoprofen self-administered in a single oral overdose are shown in Fig. 1B. The plasma concentrations of loxoprofen and its reduced *trans-*alcohol form in the current patient were 52 and 24 μg/mL, 3.7 and 2.3 μg/mL, 0.81 and 0.54 μg/mL, and 0.015 and 0.011 μg/mL at 4, 26, 50, and 121 h, respectively, after an oral overdose of 6000 mg.

We also report the results of in silico pharmacokinetic modeling of plasma concentrations. Based on the reported human blood concentrations after 24 healthy volunteers were orally administered a normal therapeutic dose [4, 14] (shown in Fig. 1A), a simple loxoprofen PBPK model consisting of gut, liver, kidney, and central and peripheral compartments was set up as described previously [12, 13, 15]. The initial values for the fraction absorbed × intestinal availability (*F*_a_·*F*_g_) and hepatic clearance (*CL*_h_) were estimated from the elimination constants in empirical one-compartment models. As input parameters for the PBPK model, the absorption rate constant (*k*_a_), volume of the systemic circulation (*V*_1_), and hepatic intrinsic clearance (*CL*_h,int_) values, with standard deviations, were determined by fitting using nonlinear regression analyses; these final parameters are shown in Table 2. The resulting system of differential equations was solved to obtain the concentrations of the substrate and its metabolite (indicated with subscript *m*) for the overdosed patient in this study:

$$ \frac{d{X}_g}{dt}=-{k}_a\cdotp {X}_g\  where\  at\ t=0,{X}_g(0)=F\mathrm{a}\cdotp F\mathrm{g}\cdotp dose $$

$$ {V}_h\frac{d{C}_h}{dt}={k}_a\cdotp {X}_g-\frac{Q_h\cdotp {C}_h\cdotp {R}_b}{K_{p,h}}-{CL}_{h,\mathit{\operatorname{int}}}\cdotp \frac{C_h}{K_{p,h}}\cdotp {f}_{u,p}+{Q}_h\cdotp {C}_b $$

$$ {V}_1\frac{d{C}_b}{dt}=-\left({Q}_h+{Q}_r\right)\cdotp {C}_b+\frac{Q_h\cdotp {C}_h\cdotp {R}_b}{K_{p,h}}+\frac{Q_r\cdotp {C}_r\cdotp {R}_b}{K_{p,r}} $$

$$ {V}_r\frac{d{C}_r}{dt}={Q}_r\cdotp {C}_b-\frac{Q_r\cdotp {C}_r\cdotp {R}_b}{K_{p,r}}-{CL}_r\cdotp \frac{C_r}{K_{p,r}}\cdotp {f}_{u,p} $$

$$ {V}_{h,m}\frac{d{C}_{h,m}}{dt}={Q}_h\cdotp {C}_{b,m}-\frac{Q_h\cdotp {C}_{h,m}\cdotp {R}_{b,m}}{K_{p,h,m}}+{CL}_{h,\mathit{\operatorname{int}}}\cdotp \frac{C_h}{K_{p,h}}\cdotp {f}_{u,p}-{CL}_{h,\mathit{\operatorname{int}},m}\cdotp \frac{C_{h,m}}{K_{p,h,m}}\cdotp {f}_{u,p,m} $$

$$ {V}_{1,m}\frac{d{C}_{b,m}}{dt}=-\left({Q}_h+{Q}_r\right)\cdotp {C}_{b,m}+\frac{Q_h\cdotp {C}_{h,m}\cdotp {R}_{b,m}}{K_{p,h,m}}+\frac{Q_r\cdotp {C}_{r,m}\cdotp {R}_{b,m}}{K_{p,r,m}} $$

$$ {V}_{r,m}\frac{d{C}_{r,m}}{dt}={Q}_r\cdotp {C}_{b,m}-\frac{Q_r\cdotp {C}_{r,m}\cdotp {R}_{b,m}}{K_{p,r,m}}-{CL}_{r,m}\cdotp \frac{C_{r,m}}{K_{p,r,m}}\cdotp {f}_{u,p,m} $$

where *X*_g_, *V*_h_, *V*_r_, *C*_h_, *C*_r_, and *C*_b_, respectively, are the amounts of compound in the gut compartment; the liver and kidney volumes; and the hepatic, renal, and blood substrate concentrations. The blood-to-plasma concentration ratio (*R*_b_) and the liver-to-plasma and kidney-to-plasma concentration ratios (*K*_p,h_ and *K*_p,r_) of the relevant compounds were estimated from plasma unbound fraction (*f*_u,p_) and octanol–water partition coefficient (log*P*) values [11, 16, 17]. *V*_*h*_ and *V*_r_ are the liver (1.5 L) and kidney (0.28 L) volumes, and *Q*_h_/*Q*_r_ are the blood flow rates of the systemic circulation to the hepatic/renal compartments (96.6 L/h) [11].

**Table 2:** Physiological, experimental, and final calculated parameters for loxoprofen PBPK model established in this study

Parameter	Loxoprofen	Reduced *trans-*alcohol metabolite of loxoprofen
Model input parameters		
Molecular weight	246	248
Octanol–water partition coefficient	1.97	2.23
Plasma unbound fraction	0.0681	0.0559
Blood–plasma concentration ratio	0.799	0.782
Liver–plasma concentration ratio	1.15	1.49
Fraction absorbed × intestinal availability	1	–
Absorption rate constant, 1/h	6.17 ± 0.27 ^a^	–
Volume of systemic circulation, L	4.67 ± 0.17 ^a^	11.4 ± 0.3 ^a^
Hepatic intrinsic clearance, L/h	76.3 ± 0.3 ^a^	215 ± 1 ^a^
Hepatic clearance, L/h	4.93	10.7
Renal clearance, L/h	0.10	1.1
Estimated values ^c^		
C_max_ in plasma, ng/mL	6940 (0.97) ^b^	770 (0.86) ^b^
AUC in plasma, ng h/mL	11,400 (1.1)^b^	2380 (0.90) ^b^
Reported levels		
C_max_ in plasma, ng/mL ^d^	7160	896
AUC in plasma, ng h/mL ^d^	10,700	2650
Bioavailability ^d^	1	–
Urinary excretion of unchanged drug ^d^	0.02	–

The plasma unbound fraction, octanol–water partition coefficient, blood-to-plasma concentration ratio, and liver-to-plasma concentration ratio of loxoprofen and the reduced *trans-*alcohol metabolite of loxoprofen were estimated using in silico tools [15]

^a^ Data are means ± standard deviations by fitting to observed concentrations

^b^ Values in parentheses are ratios to the reported/observed values

^c^ PBPK modeled values for a virtual administration of 60 mg loxoprofen

^d^ Taken from the literature [4, 14], as shown in Fig. 1A

## Discussion and conclusions

To the best of our knowledge, no case reports are available in the international literature relating to the disturbance of consciousness caused by overdosed loxoprofen. In the present study, we investigated the association between disturbance of consciousness and the expected high blood levels of loxoprofen in the current patient. A single oral 60-mg dose of loxoprofen administered to 24 healthy Korean men resulted in average peak serum concentrations of 4.8 μg/mL at 0.5 h and 2.4 μg/mL at 0.9 h for loxoprofen and its reduced *trans*-alcohol metabolite, respectively [18]. In the current overdose case, the measured plasma concentration levels of loxoprofen were around 50 μg/mL. The clinical laboratory results (Table 1) indicate that this plasma level of loxoprofen likely does not cause hepatic impairment, but slight renal impairment was noted. Regarding the absorption phase pharmacokinetics of loxoprofen, it was assumed that loxoprofen at 100-fold the therapeutic dose was still within the linear range; this assumption was based on the fact that plasma concentrations of loxoprofen and its *trans*-alcohol metabolite 4 h after an oral overdose of 6000 mg were consistent with the simulated concentration profiles (Fig. 1B).

In contrast, the elimination phase pharmacokinetics evidently fell outside the linear range. The observed terminal elimination half-life values of 6–12 h (calculated using two data points) after the 6000-mg dose were several-fold longer than the reported normal values of 1–2 h, thereby implying dose non-linearity for loxoprofen elimination pharmacokinetics over a range of 100-fold the normal therapeutic dose in the current case (Fig. 1). It was noted in the current case that gastrointestinal symptoms, liver dysfunction, thrombocytopenia, and severe renal failure were not observed. The reasons for the delayed eliminations from plasma of loxoprofen and its *trans*-alcohol metabolite in this case are uncertain, but slight renal impairment (low eGFR values, Table 1) that developed on the second and third hospital days could be one of the causal factors. It could not be ruled out a possibility that loxoprofen might be biphasically eliminated from the plasma even in the healthy subjects in the range from several hours to 120 h. Although this case report of an overdose of 100 tablets (6000 mg) loxoprofen is considered to be the first to appear in English, the case of a woman in her thirties who took 3600 mg of loxoprofen has been reported in Japanese [19].

In hospitals, a simplified PBPK model-based simulator [20] may replace the need to routinely measure the blood levels of drugs. The present results based on drug monitoring data and pharmacokinetic predictions for the absorption and elimination phases could serve as a promising guide when setting the treatment period in cases of overdoses.

## Data Availability

All data generated or analyzed during this study are included in this published article and are also available from the corresponding author on reasonable request.

## Abbreviations

NSAID: non-steroidal anti-inflammatory drug
PBPK: physiologically based pharmacokinetic
